# Facilitating Text Production in Fourth Graders: Effects of Script-Based Knowledge and Writing Prompts

**DOI:** 10.3389/fpsyg.2022.821011

**Published:** 2022-04-01

**Authors:** Béatrice Bourdin, Michel Fayol

**Affiliations:** ^1^University of Picardie Jules Verne, Amiens, France; ^2^CRP-CPO, UR UPJV 7273, Université de Picardie Jules Verne, Amiens, France; ^3^Université Clermont Auvergne, Clermont-Ferrand, France; ^4^UMR 6024 Laboratoire de Psychologie Sociale et Cognitive (LAPSCO), Clermont-Ferrand, France

**Keywords:** translation, written production, semantic relatedness, children, writing prompts

## Abstract

This study aimed at providing evidence that prior knowledge (semantic relatedness) and its organization (scripted versus not related) prompted either through pictures alone, pictures and associated words, words only have different impacts on several components of text produced by fourth graders. The results showed that the semantic relatedness affected three dependent measures: prompt words recalled, coherence and quality of texts. The nature of the prompts impacted on planning (number of ideas) and translating (number of propositions and length of texts) processes. Findings, instructional applications, limitations, and proposals for future research are discussed.

## Introduction

Written composition is a highly complex and dynamic process encompassing several interacting subcomponents: Planning involves setting goals, retrieving ideas from memory, and organizing contents into a writing plan; translating consists of gradually constructing the text as a linear sequence of linguistic units which are ordered hierarchically by level (e.g., words, phrases, sentences, paragraphs); reviewing includes monitoring and editing the text produced so far (Hayes and Flower, 1980). Based on accumulating research that handwriting and spelling (i.e., transcription) affect the development of translation, transcription was added to the 1980 model (Berninger and Swanson, 1994). The initial Hayes and Flower’s model has been reorganized to take into account the dynamic of production, and the role of knowledge (i.e., experience) and working memory in the translation process (Hayes, 2012). More recently, Kim and her collaborators have devised and integrative and hierarchical model of developmental writing (i.e., DIEW: Direct and Indirect Effects model of Writing) encompassing multiple processes (Kim and Park, 2019; Kim, 2020; Kim and Graham, 2022). This model takes into account all the dimensions involved in the written production of texts -language, cognition, print-related skills- and, more interestingly, knowledge of the topic. According this model, these multiple dimensions have hierarchical, interactive, and dynamic relations. Among these dimensions, the knowledge of the topic contributes little to the quality of written compositions. It would be constrained by language and transcription skills for developing writers. However, the knowledge of the topic has been controlled indirectly using a questionnaire. It is possible that the impact of this factor is greater when the theme to be produced is controlled. Therefore, the main objective of the current research is to provide evidence of prior knowledge (i.e., semantic relatedness from script-like event sequences) and its organization on different components of text production in fourth graders.

The main problem facing children producing written composition concerns the coordination of composing components, while calling on only a limited pool of cognitive resources (McCutchen, 1996; Fayol et al., 2012; Olive, 2014). Composing is a goal-directed activity: most resources are devoted to the global control of the production processes, taking into account the audience, the concepts and their organization and the way the linearization could be realized. This controlled processing is slow and demands focused attention and conscious mental effort. In addition, the writer also has to bring different processes or skills to bear, such as transcribing or grammatical encoding.

Several studies have tried to reduce the costs of some components in order to make composition tasks more manageable. Some of these attempts have concentrated on improving transcription processes: increasing writing speed and improving spelling performance have led children to produce longer and better quality texts (Graham et al., 2000; Alves et al., 2016). Some other studies have been working on strategies, changing the relations between the components of the tasks and enabling self-regulated and adapted moves from parallel to serial processing (Graham et al., 2005; Limpo and Alves, 2013). Generally, all these attempts have been successful.

A third less explored possibility is to improve the knowledge base of text producers. Indeed, the more people know about the topic to be dealt with in composition, the higher the length and the quality of the texts they produce (McCutchen, 1986, with adults; Olinghouse et al., 2015 with 5th graders producing stories, persuasive papers and informational texts; Wijekumar et al. (2019), with 5th graders on a persuasive writing task) and the less the mental effort they have to invest in the composition process (Kellogg, 2004). Berninger et al. (1996) reported a clear link between knowledge and writing strategies in a study of graduate students working in a novel domain. Without the benefit of topic knowledge and a familiar genre, skilled writers can lose access to part of their knowledge and skills in long term memory and resort to less mature strategies to cope with writing demands. Composition performance is thus highly dependent on the conceptual and declarative knowledge about representations of the evoked situations.

To illustrate, most children and adults know what is happening in a birthday party, in going to the swimming pool, in being in a restaurant, in making cookies, in visiting a museum, and so on (Ornstein and Haden, 2001). This scripted knowledge is determined by familiar activities and recurring events in the life, which are very similar in children, adolescents and adults. It helps people to understand what is going on in real life and in reading texts, and it also guides text production. The predictability of the ordered events in scripts for familiar routines facilitates text production (Hudson and Shapiro, 1991). Writers may draw upon this scripted knowledge, which reduces mental effort, leaving more working memory capacity to elaborate the chain in a script for familiar knowledge for situations, routines, or event. Bourdin and Fayol (2002) used semantically related or unrelated items from familiar scripts with adults and found that the texts were less elaborated and less organized ideas when the events evoked through the supplied series of prompts (i.e., words) were difficult to link in a coherent way (i.e., they did not form a script). Following the same line of reasoning, we gave children a series of items from scripted events presented in either their canonical order within a script (related) or in a mixed way (unrelated). Items related to a single script would help children to produce a coherent sequence. By contrast, writers would find it difficult to elaborate an event chain from items unrelated to a single script.

Not all cognitive-linguistic production draws on verbal stimuli such as words. Even if the nature of the script is held constant, the cognitive representation that is accessed during composition may be nonverbal. Several results provide evidence that pictures or drawings are better prompts than words to help recall or understanding of texts (Levie and Lentz, 1982). According to these authors, pictures facilitate the elaboration of the mental model of the situation referred to. We could thus expect that pictures as prompts would help improve text production as well. Combining pictures with the corresponding words would improve text production even more because words are elements of the translation process. Their presence would facilitate lexical selection and sentence production, and thus alleviate the load of text production.

The current study examined two research questions. The first question was: Do script-like conceptual knowledge facilitates text composition through alleviating the burden of text production? To answer this question, we compared the impact on composition characteristics of manipulating the material provided to the writers to support their composition: series of words and/or pictures corresponding to routine activities (e.g., going to the swimming pool) presented in the conventional or in pseudo-random order. We test the hypothesis that giving children a series of items semantically related to familiar scripts would facilitate the production of coherent and good quality texts. The second question was: to what extent the writing prompts: words and/or pictures corresponding to the sequence of events have an impact on the characteristics of the texts produced by children? We argue that prompts using pictures alone or a mix of words and pictures would more facilitate text production in children than words alone. In this perspective, groups of about 10 children were provided auditory and visually series of either 8 words alone, 8 pictures alone, 8 pictures and words corresponding to script-like or to pseudorandom event sequences. After this presentation, they were asked to compose texts including the words in the set.

## Materials and Methods

### Experimental Design

Two variables were manipulated in this experiment: semantic relatedness (series of items related versus not related to familiar routine activities in scripts); and writing prompt: words (W), pictures (P), pictures and words (PW).

### Participants

Sixty-four fourth graders (mean age = 9.4 years, range: 8.7 – 10.2, 36 girls) from several villages in Auvergne Rhone-Alpes, whose parents granted informed consent, agreed to participate and so did their teachers. The study was conducted in compliance with state French and European ethical norms related to research with human participants. The 64 children were randomly assigned to 6 experimental groups: three conditions (Words, Pictures, Pictures+ Words) x 2 (related versus unrelated items) of 10 or 11 participants each.

All pupils have been collectively submitted to a series of tests intended to verify that the six groups did not differ on several important dimensions related to text production. Based on the work of Berninger and Fuller (1992), the writing speed has been evaluated by asking children to write the alphabet in cursive writing, as quickly but as legibly as possible. To assess their spelling ability, we used the “Corbeau” (The crow) dictation from the L2MA from Chevrie-Muller et al. (1997). Finally, we tested their short-term verbal memory using a digit recall task. The digits are taken from the BALE test (Jacquier-Roux et al., 2010). All these tests have been adapted to collective presentations and written responses. The 6 groups did not differ significantly in these tests (cf Table 1).

**TABLE 1:** Means, standard deviations and ANOVA results for each group*.

	Staff	Age (years)	Short-term memory	Writing Speed	Spelling ability
Group 1 (R/P)	11	9.4	36.1 (8.5)	9.6 (1.96)	31.8 (6.7)
Group 2 (U/P)	11	9.4	37.6 (7.8)	10.5 (2.8)	35.9 (3.4)
Group 3 (R/W+P)	10	9.4	34.6 (7.6)	9.4 (2.2)	32.5 (6.7)
Group 4 (U/W+P)	10	9.2	38.8 (8.7)	12.3 (3.0)	34.0 (5.2)
Group 5 (R/W)	11	9.5	38.3 (6.1)	11.9 (2.8)	36.2 (6.9)
Group 6 (U/W)	11	9.3	31.5 (6.2)	11.5 (1.9)	35.2 (4.9)
Anova		F < 1	F(1,5) = 1.68, ns	F(1,5) = 2.42, ns	F < 1

*R: related, U: unrelated, P: pictures, W: words.

### Stimuli and Material

The material and procedures in Bourdin and Fayol (2002) were adapted for children. Sixty-four frequent bisyllabic words were used (frequency was 4,128 per 100 million from the Trésor de la Langue Française; IMBS, 1971). These words were subdivided into 8 sets of 8 words each (Table 2). Each set corresponded to a script for a variety of procedural domains (going to the sea; going to the hairdresser; going to school; going to the supermarket; going to the restaurant; going to the doctor; going to the cinema; getting up in the morning) (Schank and Abelson, 1977). The nouns corresponding to the core elements of the scripts were used in this experiment. The 64 words were divided into eight lists of eight words in each of two sets. The 8 words lists in the first set corresponded to the scripts, resulting in related words. Each of the 8 words lists in the second set included 8 words, one from each different script, resulting in unrelated words. The mean frequency of the words assigned to the “related” vs. “unrelated” conditions did not differ (Fs < 1).

**TABLE 2:** Lists of semantically related and unrelated words.

Lists of semantically related words			
***List 1***	***List 2***	***List 3***	***List 4***
*Aller à la mer*	*Aller chez le coiffeur*	*Aller à l’école*	*Aller au supermarché*
(going to the sea)	(going to the hairdresser)	(going to school)	(going to the supermarket)
sable (sand)	cheveux (hair)	classe (school)	client (customer)
galet (pebble)	ciseaux (scissor)	tableau (blackboard)	chariot (truck)
vague (wave)	blouse (smock)	leçon (lesson)	rayon (shelf)
marée (tide)	salon (salon)	dictée (dictation)	course (shopping)
plage (beach)	peigne (comb)	crayon (pencil)	viande (meat)
algue (alga)	brosse (hairbrush)	cahier (notebook)	poisson (fish)
pêche (fishing)	miroir (mirror)	calcul (arithmetic)	bonbon (sweet)
bateau (boat)	serviette (towel)	classeur (file)	caisse (cashdesk)
***Liste 5***	***List 6***	***List 7***	***List 8***
*Aller au restaurant*	*Aller chez le docteur*	*Aller au cinéma*	*Se préparer le matin*
(going to the restaurant)	(going to the doctor)	(going to the cinema)	(getting ready in the morning)
plateau (tray)	docteur (doctor)	caisse (cashdesk)	habit (clothes)
assiette (plate)	virus (virus)	entrée (entrance)	réveil (alarm)
menu (menu)	gorge (throat)	salle (cinema)	manteau (coat)
serveur (waiter)	cachet (tablet)	ticket (ticket)	leçon (lesson)
café (coffee)	sirop (syrup)	glace (ice cream)	tartine (slice)
entrée (first course)	rhume (cold)	fauteuil (chair)	douche (shower)
dessert (dessert)	patient (patient)	écran (screen)	café (coffee)
repas (meal)	bureau (office)	billet (ticket)	peigner (comb)
**Lists of semantically unrelated words**			
***List 1***	***List 2***	***List 3***	***List 4***
sable (sand)	plateau (tray)	écran (screen)	algue (algae)
fauteuil (chair)	cachet (tablet)	pêche (fishing)	caisse (cashdesk)
chariot (truck)	marée (tide)	leçon (lesson)	dictée (dictation)
brosse (hairbrush)	ciseaux (scissor)	bureau (office)	leçon (lesson)
tartine (slice)	client (customer)	bonbon (sweet)	peigne (comb)
docteur (doctor)	glace (ice cream)	miroir (mirror)	billet (ticket)
menu (menu)	classeur (file)	douche (shower)	repas (meal)
tableau (blackboard)	réveil (alarm)	café (coffee)	gorge (throat)
***List 5***	***List 6***	***List 7***	***List 8***
assiette (plate)	classe (school)	poisson (fish)	patient (patient)
rayon (shelf)	plage (beach)	habit (clothes)	viande (meat)
sirop (syrup)	salon (salon)	rhume (cold)	crayon (pencil)
cahier (notebook)	ticket (ticket)	blouse (smock)	serviette (towel)
bateau (boat)	manteau (coat)	entrée (first course)	galet (pebble)
entrée (entrance)	serveur (waiter)	calcul (arithmetic)	peigner (comb)
cheveux (hair)	course (shopping)	caisse (cashdesk)	dessert (dessert)
café (coffee)	virus (virus)	vague (wave)	salle (cinema)

In order to illustrate each set with pictures (see Table 3 for an example), we selected all pictures associated with the words (Deloche and Hannequin, 1997; Bonin et al., 2003). We changed a small number of words (e.g., virus - > microbe) to have a better match between nouns and pictures; then we verified that the mean word frequencies still did not differ based on the Manulex database (Lété et al., 2004). Second, the three writing prompts and two semantic relatedness conditions (related and unrelated) were organized in 48 slide-shows of 8 slides each: one slide-show for each condition (e.g., 8 for Related/Pictures condition, 8 for Related/Pictures+Words, etc.).

**TABLE 3:** Example of pictures for the list “going to the hairdresser.”

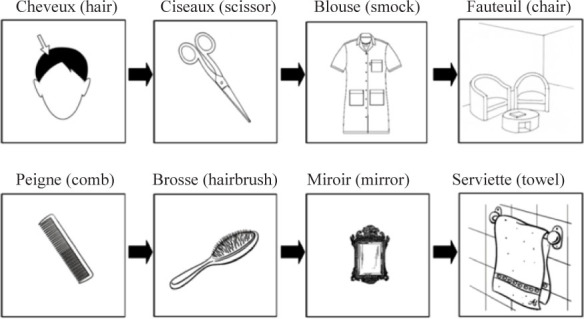

### Procedure

The data have been collected through several steps by two experimenters rigorously trained who were present in all classes during the passations. They trained the children according a written protocol in order to ensure that the design model was the same in all conditions.

**FIGURE 1:**
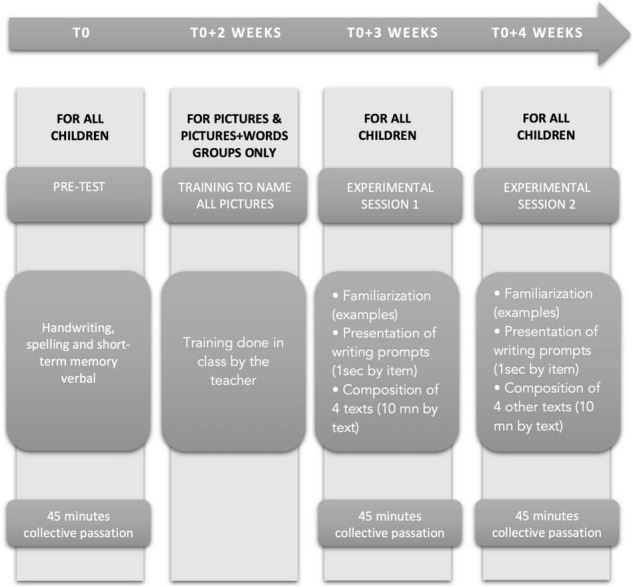
General design of the procedure used.

Firstly, at the beginning of the school year, all children were submitted to the tests aimed at determining individual performances in handwriting, spelling, and short-term memory verbal. Secondly, some weeks later, they were trained to name all the pictures and to read all the words. Thirdly, some days before the experiment, children were trained with lists of words or images different from those used in the experiment, so that they knew exactly what was being asked of them. They were provided several examples of slide-shows different from those used in the experiment under the different conditions (i.e., words, pictures, words, and pictures) from which they were invited to compose texts. For each text production, they were provided prompts (words, picture, pictures and words), invited to read the words and to examine the pictures when present. They were explained not to care about word or pictures order: the important goal was to produce texts. Fourthly, the prompts were removed and they had to write the text corresponding to each set of items. Each group participated in two 45 min sessions of text production (4 texts in each session), one per week (see Figure 1 for the general design of the procedure used). No time constraint, or length of texts was imposed on the children.

### Scoring

Two scores were computed related to recalled words: First, the absolute number of words recalled from the lists (1 point for every word); and second, the number of words included in semantically and grammatically correct sentences or propositions. Text length (one point per word) was also computed. Then two independent judges assessed the number of semantically and grammatically correct sentences, the number of propositions, the number of ideas, and the level of coherence. Finally, two other judges working independently evaluated the global quality of texts using a grid elaborated from previous experimental studies (Olive and Piolat, 2003). The grid included 5 criteria, each scored on a 5-point scale (very low, low, average, good, very good) (global score from 0 to 25 max.): Coherence of ideas and structure of the whole text; cohesion (connectives and punctuation); syntax (verbal tenses, use of pronouns, syntactic structures); lexical spelling; and grammatical spelling. The judges first independently scored each text and then discussed each disagreement in order to come to an agreement on the score.

### Data Analyses

As our data not always meet the assumptions of normality, and given our small sample size, all the data have been submitted to non parametrical analyses with The Jamovi Project (2021). We used Man-Whitney U test to determine the material effect (related vs unrelated) and compare means in related and unrelated conditions for each of the variables considered (number of words recalled, number of ideas, length of texts, level of coherence, number of correct propositions and global evaluation). We conducted a one-way anova for independent measures in order to evaluate the impact of writing prompt (W, P, PW), followed by Dwass-Steel-Critchlow-Fligner (DSCF) pairwise comparisons.

## Results

### Prompt Words Recalled

The number of words recalled did not differ as a function of semantic relatedness (related = 4.36; unrelated = 4.02), (U = 414, *p* = 0.095, Cohen’s d = 0.191). The effect of prompts was not significant (W = 4.26; *P* = 4.24; PW = 4.07), (X^2^ = 0.556, df = 2, *p* = 0.757, ε^2^ = 0.009). No pairwise comparisons were significant (P/PW = −0.749, *p* = 0.857; P/W: W = 0.285, *p* = 0.978; PW/W: W = 0.997, *p* = 0.761).

### Ideas and Organization

The number of ideas expressed did not differ between texts composed from related (5.39) or unrelated (5.05) series of items, (U = 500, *p* = 0.439, Cohen’s *d* = 0.023). By contrast, this number differed as a function of the prompts (X^2^ = 11.2, df = 2, *p* = 0.004, ε^2^ = 0.179). Planned comparisons showed that texts produced from pictures alone (W = −4.42, *p* = 0.005) or associated to words (W = −3.57, *p* = 0.031) included more ideas than those composed from words only (respectively: 5.80, 5.59, and 4.32). No significant difference was observed between PW and P conditions, (W = −1.07, *p* = 0.73).

About coherence, texts were judged as more coherent when produced from related lists (3.79) rather than unrelated ones (2.58), (U = 89.5, *p* < 0.001, Cohen’s *d* = 0.825). There was no effect of prompts (W = 3.23; *P* = 3.34; PW = 3), (X^2^ = 1.82, df = 2, *p* = 0.403, ε^2^ = 0,029). No pairwise comparisons were significant (P/PW: W = −1.784, *p* = 0.417; P/W: W = −0.285, *p* = 0.978; PW/W: W = 1.462, *p* = 0.556).

### Performances in Translation

The mean length of the texts varied as a function of the provided prompts (X^2^ = 8.14, df = 2, *p* = 0.017, ε^2^ = 0.129) but not as a function of relatedness (U = 470, *p* = 0.289, Cohen’s *d* = 0.082), (related = 30.7 words, unrelated = 27.4 words). Planned comparisons showed that texts produced from pictures alone (W = −3.829, *p* = 0.019) or associated to words (W = −3.087, *p* = 0.074) included more words than those composed from words only (respectively: 30.7, 31.5, and 25.1). No significant difference was observed between PW and P conditions, (W = −0.267, *p* = 0.981).

The number of words included in semantically and grammatically acceptable sentences varied as a function of relatedness (U = 296, *p* = 0.002, Cohen’s *d* = 0.422; 4.17 under related condition versus 3.38 under unrelated condition). The effect of prompts was not significant (W = 3.57; P = 4; PW = 3.75), (X^2^ = 2.05, df = 2, *p* = 0.358, ε^2^ = 0.033). No pairwise comparisons were significant (P/PW: W = −0.517, *p* = 0.929; P/W: W = −2.194, *p* = 0.267; PW/W: W = −1.097, *p* = 0.718).

The number of syntactically correct sentences depended on the prompts (X^2^ = 12.4, df = 2, *p* < 0.002, ε^2^ = 0.330) but not on relatedness (U = 440, *p* = 0.17, Cohen’s *d* = 0.142). Planned comparisons showed that texts produced from pictures alone (W = −4.54, *p* = 0.004) or associated to words (W = −3.99, *p* = 0.013) included more propositions than those composed from words only (respectively: 4.85, 4.79, and 3.52). No significant difference was observed between PW and P conditions, (W = −0.481, *p* = 0.938).

### Global Evaluation

The total score (max 25) varied significantly as a function of semantic relatedness: texts composed from related series of items (16.8) were considered as productions of better quality than those written from unrelated series (15.6), (U = 312, *p* = 0.004, Cohen’s *d* = 0.392). There was no effect of the different prompts (W = 16.8; P = 16.1; PW = 15.7), (X^2^ = 3.86, df = 2, *p* = 0.145, ε^2^ = 0.061). No pairwise comparisons were significant (P/PW: W = −1.62, *p* = 0.485; P/W: W = 1.21, *p* = 0.668; PW/W: W = 2.69, *p* = 0.138).

## Discussion

The research aimed at answering two questions. First, do children write better texts when they have sets of items corresponding to the usual course of events (i.e., semantically related script-like sequences) rather than when these items come from different sequences of events (semantically unrelated). Second, do the different types of prompts (words, images, etc.) have the same effect on the composition of the texts or do some favor certain dimensions of the production more or less?

The two manipulated variables – semantic relatedness (related versus unrelated series of items) on the one hand; prompts (words or pictures alone or pictures with words) on the other hand – had quite different effects of the two main dimensions of text composition: planning and organizing the content; translating (i.e., turning ideas into words, sentences, and larger units of discourse).

Semantic relatedness impacted mainly the global quality of the texts, the coherence of the whole set of ideas and the number of prompt words inserted into semantically and grammatically correct sentences. The effect size for this factor varied from medium to large as a function of dependent measures, attesting to the concrete importance of semantic relatedness. This result is in agreement with previous results from Olinghouse et al. (2015), Kim (2020) who reported that children and adults generated more content and better quality texts when composing about familiar topics, compared with unfamiliar one. Extensive well organized knowledge enables better and faster access to memorized facts and words, both making easier and more coherent text composition and alleviating the load of hanwriting and spelling, hence improving the quality of sentences. However, our results do not allow us to verify whether the effect of topic knowledge is mediated by other factors, such as transcription for example. Our sample size is insufficient to conduct statistical analyses that would allow us to answer this question.

By contrast, the prompts, pictures and/or words, impacted essentially the translation process. Indeed, children produced more ideas from picture and/or words than from words alone, and more interestingly adding words to pictures did not improve the number of ideas. They also produced more syntactically correct sentences. Pictures more than words helped children to retrieve or reconstruct ideas about the script frame. This is a new result, and it is a bit amazing because providing scripts under the word prompts (instead of the picture prompts) could have helped chidren to elaborate sentences. On the contrary, even when providing words in addition to pictures, the syntactic forms were not improved. This result is in line with Baadte and Meinhardt-Iniac (2019) who tested whether semantic relatedness (e.g., pairs of stimuli, related - padlock-key- versus unrelated - lemon-piano) between to-be-remembered items and pictorial vs. verbal item presentation affected associative recall in adolescents and adults. They reported a relatedness superiority effect for picture-picture pairs over word-word and even picture-word, exactly as we found in written production from script sequences. Children find it more difficult to retrieve ideas from words alone, even when those words come from familiar event sequences. However, as claimed by Baadte and Meinhardt-Iniac, a gap in understanding which cognitive and individual variables may contribute to picture superiority. However, it is important to remember that in our study the prompts were removed when children composed. The results might have been different if the prompts had been available during texts production.

The absence of significant interaction between item relatedness and prompt modality suggests that two different and independent processes are in play, one dealing with event representations sequences, and the other operating translation from ideas to words, sentences and texts. In the present experiment, these two processes seem to operate independently of each other and the conditions of data collection do not allow to determine how they are coordinated. This dissociation of effects between higher-order dimensions (i.e., semantic relatedness impacting writing quality and coherence) and the impact of prompts onto translation processes observed in 4th graders’ text production is in agreement with the recent integrative model developed by Kim and Graham (2022) and explored in 2nd graders’ compositions. Additional longitudinal studies would be needed to explore in more detail whether the results found with younger and older pupils would confirm the data reposted here.

Our findings should be considered in light of some limitations. First our sudy have small number of children per experimental condition (about ten). It has been difficult to obtain 8 groups of pupils matched on several dimensions. Future studies should try to replicate our findings with a larger sample size. Moreover, the generalizability of our results is limited to 4th graders. Future studies are needed to replicate and extend the present study with both younger and older pupils, and adults. A second limitation relating to this work concerns the evaluation of the texts. It would be useful to have several judges and to estimate their agreements or divergences. A third limitation is the possible confound between relatedness/unrelatedness and difficulty: of course, scripts correspond to previous knowledge already available and easily activated to perform the composition task. By contrast, unrelated sequences are *a priori* less coherent and children have to seek and establish the coherence between events. This task is most probably difficult for 4th graders who still lack metacognitive skills. Such a situation of text production should be replicated with 6th to 8th participants.

### Theoretical and Instructional Relevance

These results have both theoretical and pedagogical relevance. The most important objective is to promote the development of efficient text production in elementary pupils. A recent meta-analysis shows than a multi-component instructional approach is more efficience than instructional approaches that target one specific skill (e.g., transcription), (Kim et al., 2021). Our results also provide elements of intervention to improve student’s written production. First, previous knowledge and its organization plays an important role in the elaboration of a coherent mental model of event sequences. Until now this role has been a bit underestimated in studying and teaching text production. More research is needed to determine how helping children to rely on previous knowledge or providing them information before writing could improve their text production and help them to progressively lead them to a capability for autonomous production of texts (cf Alves, 2019). Second, the positive impact of pictures over words suggests that even in the 4th grade retrieving words and their associations with facts and events remains costly. Providing pictures is helpful, most probably because it helps children to elaborate at least partial representations that, in turn, facilitate word and sentence productions, as observed in fluency and sentence production. As a consequence, it could be useful to verify that providing pictures to the younger pupils could facilitate their text productions, and progressively diminishing the frequency of such scaffolds to focus on words. Moreover, real-time sudies would be needed to explore this question. At the beginning of elementary school, teachers could provide non-verbal prompts (pictures) to help pupils improve the elaboration of an integrated mental model before translation or provide both non-verbal and verbal prompts (words) to support the translation process.

## Data Availability Statement

The raw data supporting the conclusions of this article will be made available by the authors, without undue reservation.

## Ethics Statement

Ethical review and approval were not required for the study on human participants in accordance with the local legislation and institutional requirements. Written informed consent to participate in this study was provided by the participants’ parents or the participants’ legal guardian/next of kin.

## Author Contributions

Both authors contributed to the article and approved the submitted version.

## Conflict of Interest

The authors declare that the research was conducted in the absence of any commercial or financial relationships that could be construed as a potential conflict of interest.

## Publisher’s Note

All claims expressed in this article are solely those of the authors and do not necessarily represent those of their affiliated organizations, or those of the publisher, the editors and the reviewers. Any product that may be evaluated in this article, or claim that may be made by its manufacturer, is not guaranteed or endorsed by the publisher.
